# A cognitive fingerprint in human random number generation

**DOI:** 10.1038/s41598-021-98315-y

**Published:** 2021-10-12

**Authors:** Marc-André Schulz, Sebastian Baier, Benjamin Timmermann, Danilo Bzdok, Karsten Witt

**Affiliations:** 1 Department of Psychiatry and Psychotherapy, Charité-Universitätsmedizin Berlin, Berlin, Germany; 2 Department of Neurology, Christian-Albrecht-University Kiel, Kiel, Germany; 3 Department of Biomedical Engineering, Faculty of Medicine, McGill University, Montreal, Canada; 4 Mila-Quebec AI Institute, Montreal, Canada; 5 Department of Neurology and Research Center Neurosensory Science, Carl Von Ossietzky University, Oldenburg, Germany

**Keywords:** Neuroscience, Psychology

## Abstract

Is the cognitive process of random number generation implemented via person-specific strategies corresponding to highly individual random generation behaviour? We examined random number sequences of 115 healthy participants and developed a method to quantify the similarity between two number sequences on the basis of Damerau and Levenshtein’s edit distance. “Same-author” and “different author” sequence pairs could be distinguished (96.5% AUC) based on 300 pseudo-random digits alone. We show that this phenomenon is driven by individual preference and inhibition of patterns and stays constant over a period of 1 week, forming a *cognitive fingerprint*.

## Introduction

Most work in the neurosciences collapses data from multiple subjects to obtain robust statistical results, which ignores that even in healthy subjects brain structure and function are known to be highly variable^1^. In this study we explore the question of whether cognitive processes may bear highly individual strategies—i.e. “Can we identify single individuals based on how they think?”. We address the general question of interindividual differences in the specific context of human random number generation and search for a *cognitive fingerprint* in random sequences.

Variations in executive function are reflected in the characteristic ways in which humans deviate from mathematical randomness^2^. Based on this premise, random number generation has previously been used to investigate cognitive changes in brain disorders, such as in Parkinson's disease and schizophrenia^3,4^.

**Figure 1:**
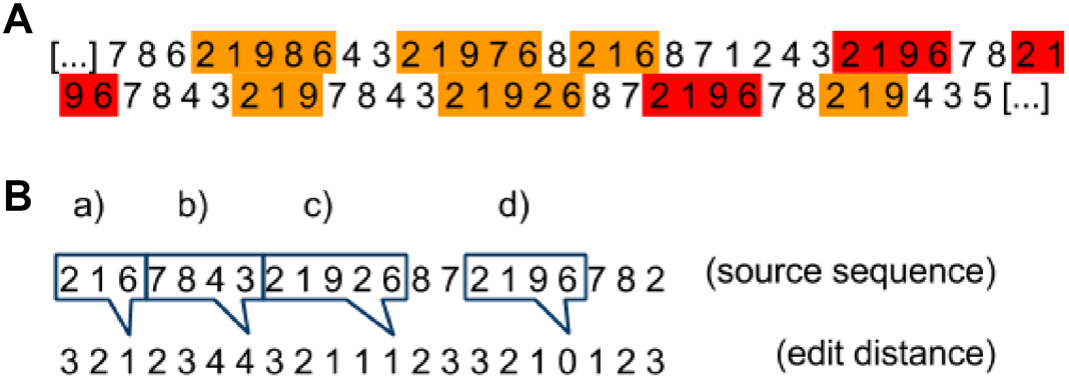
Demonstration of the pattern based approach. (**A**) In this sequence, the pattern (2, 1, 9, 6), marked in red, is predominant. Variations of this pattern are marked in orange. (**B**) Demonstrates the concept of the edit distance according to Damerau–Levenshtein. The edit distance indicates the number of edit operations necessary to convert the humanly generated random number sequence at any position into a given pattern. A distance of 0 marks a perfect match (d). At a distance of 1, one edit operation is needed to convert the sequence string into the pattern (a: deletion, c: insertion). If the patterns do not match to the given string of the sequence, up to 4 edit operations are needed. Therefore the score is 4 (b). The inverse numbers of the edit operations are added up and this score represents the mathematical “affinity” of a given pattern to the humanly generated random sequence with a lower score for patterns with diminished “affinity” to the original sequence. Reproduced from^9^.

Human-generated pseudorandom sequences have been repeatedly found to exhibit strong regularities: cycling of response alternatives, a tendency to count in increments of one, suppression of response repetitions, and others^5–8^. A general way to characterize these sequences is by reference to their n-gram frequencies (i.e., n-tuples are henceforth called “patterns”). In our previous work^9^ we demonstrated that this characterization can be improved by taking into account variations of patterns: humans apparently have a tendency to reuse certain predominant patterns and to obscure these recurring themes by implementing minor changes (i.e., variations) within the respective pattern (Fig. 1a).

To measure the prevalence of these variations, we borrowed a technique from computer science called “edit-distance”. The edit-distance quantifies the “distance” between two strings: the minimum number of operations needed to transform one string into the other. For our use case we chose the Damerau–Levenshtein distance^10^. This metric considers the number of insertions, deletions, substitutions, and transposition of adjacent characters (Fig. 1b). In particular, we introduced the “score” (Damerau–Levenshtein Score, DLS) function s(m, z), which takes a sequence (z) and a pattern (m) as input and returns a real number. The score of a pattern is calculated by computing the Damerau–Levenshtein distance of the pattern to the corresponding part of the random sequence on every position of the human generated random number sequence. The sum of the inverses of the distances (each incremented by one to avoid dividing by zero), divided by the sequence length, returns the score of the pattern on the random sequence. The efficacy of this approach was demonstrated by predicting the pseudorandom sequences based on the sequence's immediate history. When predicting one subject’s sequence, predictions based on statistical information from that same subject yielded a higher success rate than predictions based on statistical information from a different subject^9^. This effect motivates the present study.

To summarize, the random generation process can be characterized by certain predominant patterns (n-tuples, e.g. “4-3-2-1” is a length 4 pattern). These occur in different variations (“3-4-2-1” is a variation of the former) repeatedly throughout the sequence^9^. Variation-tolerant search based on the Damerau–Levenshtein distance measures the prevalence of a pattern in a sequence and identifies predominant patterns. Knowledge of the predominant patterns allows predicting individual upcoming digits in the running sequence.

## Methods

130 healthy adults (53 male, aged 35.3 years (SD = 15.6)) participated in the study. Subjects were recruited from the university, as well as friends and family of students, of the Christian Albrecht University Kiel, Germany. All participants reported that they were in good health and without any history of neurologic or psychiatric disease. They had not taken any medication or consumed any illicit drugs in the 3 months before the study. Cognitive disturbances (Mini Mental Status Examination < 26 points^11^ and depressive mood (Beck Depression Inventory > 15) served as exclusion criteria. The study procedure was approved by the local ethics committee of the Christian Albrecht University and subjects gave written informed consent before participating in the study. All methods were performed in accordance with the relevant guidelines and regulations.

The Random Generation Task was administered in a standardized procedure based on Towse and Cheshire^12^: participants were asked to vocally produce a random series of numbers from the range 1–9 (inclusive) at a pace of 1 Hz (metronome). The concept of randomness was explained using both the analogy of a fair dice and the analogy of repeatedly drawing digits from a hat (see supplement material 1). The criteria (a) equal distribution of numbers, (b) independence of responses (c) unpredictability and absence of patterns and algorithms, were explicitly mentioned. A practice run ensured that participants understood the instructions. Deviations from Towse and Cheshire's^12^ instructions and their underlying reasoning are also detailed in the supplementary material 1. Subjects generated two sequences of 300 digits with a 40 min pause in between, during which neuropsychological testing (MMSE, BDI) took place. 20 subjects generated an additional sequence after 1 week. Of the 130 subjects, 3 were excluded from further analysis for not maintaining a uniform digit distribution (> 3.5 $\sigma$ outlier). 12 were excluded for taking excessively long (> 15 s) pauses during testing. In sum, the data set analyzed includes 115 healthy non-depressed cognitive intact participants.

For each sequence we computed the Damerau–Levenshtein scores for all patterns of length 1–6, as previous research indicated that effects are limited to that range^9^. The score-function computes the prevalence of a pattern in a sequence. With this function, sequences can be characterized by reference to their pattern-scores. Any random sequence can be considered a point in a 9^n^ dimensional (9^n^ length-n variations if 9 digits) vector space. The euclidean distance of these points gives a measure of their similarity. To avoid that a few strategy changes could dominate the measure we standardize the scores and map the pattern-wise differences with the tanh-function. This effectively down-weights the (both positive and negative) outliers. Intuitively, the DLS distance represents the aggregate difference in pattern frequencies between two sequences, allowing for insertions, deletions, and transpositions.

$$\begin{aligned} & d_{n} (z_{1} ,z_{2} ) = \sqrt {\sum _{{i = 1}}^{{9^{n} }} tanh^{2} (s_{i} (z_{1} ) - s_{i} (z_{2} ))} \;or\;rather\;d(z_{1} ,z_{2} ) = |tanh(\overrightarrow {{s_{i} }} (z_{1} ) - \overrightarrow {{s_{i} }} (z_{2} ))| \\ & instead\;of\;the\;native \\ & d_{n} (z_{1} ,z_{2} ) = \sqrt {\sum _{{i = 1}}^{{9^{n} }} (s_{i} (z_{1} ) - s_{i} (z_{2} ))^{2} } \;or\;rather\;d(z_{1} ,z_{2} ) = |\overrightarrow {{s_{i} }} (z_{1} ) - \overrightarrow {{s_{i} }} (z_{2} )| \\ & with\;d:distance,\;z_{{1/2}} :sequences,\;s_{i} (z):score\;of\;i^{{th}} \;pattern\;on\;sequence\;z \\ \end{aligned}$$

We excluded digit repeats (“11”, “555”) from the pattern-vector space because subjects have trouble judging the probabilities of rare patterns and tend to use them inconsistently^13^. Based on this approach, we computed the distances for all pairs of sequences, separately for each pattern length. Additionally, we computed RNGT statistics Redundancy (quantifying equality of response usage) and Run-Ups (quantifying seriation, i.e. the tendency to “count”) as detailed by Towse and Neil^7^.

## Results

### Sanity check: RNGT statistics, practice and fatigue effects

The collected pseudorandom sequences corresponded to expectations in key RNGT statistics Redundancy and Run-Ups (Table 1). For both indices the data were distributed approximately log-normal. In contrast to earlier studies^14,15^, both indices exhibited statistically significant differences between timepoints, possibly fatigue or practice effects^16^. The occurrence of fatigue or practice effects is not a problem for the present study: as we attempt to show invariance in the human random number generator, such effects can only incur underestimation of the true effect size.

**Table 1:** Data correspond to expectations in key RNGT statistics^7^ but show practice or fatigue effects.

	Sequence one		Sequence two		
	MEAN ± SD	KS	MEAN ± SD	KS	Paired t-test
Redundancy	0.008 ± 0.006	D = 0.04; p = 0.99	0.010 ± 0.007	D = 0.07; p = 0.68	t = -4.20; p < 0.001
Runs	1.71 ± 0.23	D = 0.06; p = 0.77	1.58 ± 0.19	D = 0.05; p = 0.91	t = 9.64: p < 0.001

KS refers to Kolmogorov–Smirnov test of the empirical distributions against a log-normal distribution.

### Identification

**Figure 2:**
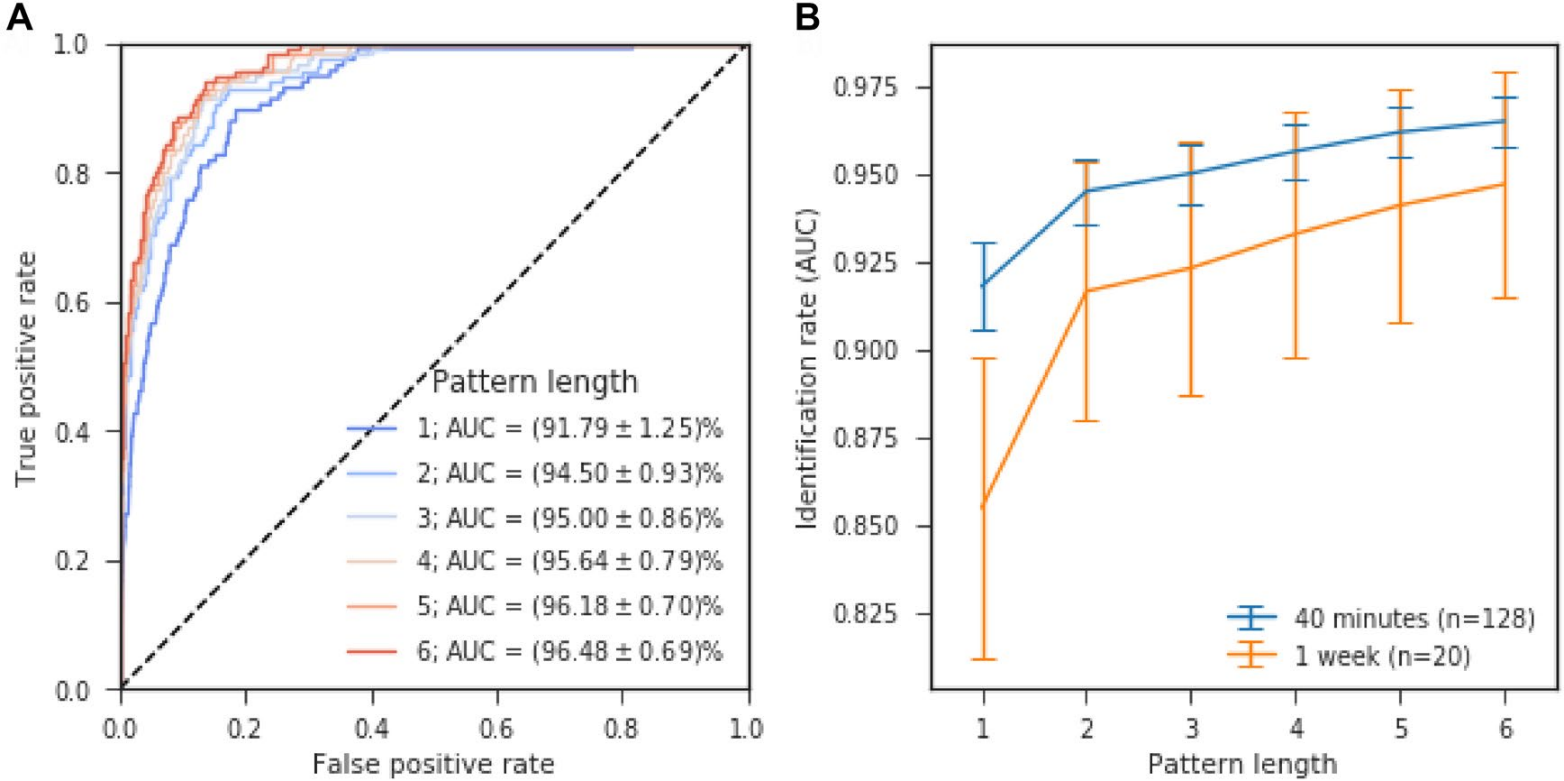
Long patterns drive identification performance. The Damerau-Levenshtein approach to quantify the similarity between two sequences was used to distinguish “same-author” and “different author” sequence pairs. (**A**) Receiver operating characteristic for the RNG-based classifier. (**B**) Identification performance (AUC) in relation to pattern-length for sequences that were generated 40 min or 1 week apart. Error bars indicate jackknife-estimated standard error of the mean.

The introduced DLS distance measure can be used to classify sequence pairs as a “match” (both sequences originate from the same subject) or “non-match” (sequences originate from different subjects) by introducing a discrimination threshold. The receiver operating characteristic (Fig. 2A) illustrates the performance of this simple classifier when its discrimination threshold is varied. Classifier performance (area under the curve, AUC) indicates a good classification performance based on single digit preferences ($91.79\%\pm 1.$25). Classifier performance increased with pattern-length (jackknife resampled linear model, z = 4.67, p < 0.001). Performance reached $(96.48\pm 0.69)\mathrm{\%}$ for length-6 patterns, a significant improvement over single-digit based prediction (p < 0.01). Note that this approach has no free parameters, which would have to be chosen subjectively by the investigators.

### Non-DLS baseline

To characterize the role of edit operations (insertion, substitutions or deletions) for our results, we additionally calculated identification performance (AUC) based on exact matches only, that is without tolerance to edit operations (non-DLS). For a pattern length of one, identification performance is identical by design. For a pattern length of two, DLS and non-DLS approaches achieved comparable results (DLS 94.50% vs non-DLS 94.43%). However, for all longer patterns, the non-DLS performance degraded, down to 84.30% at length 3, and down to the level of a random guess at length 6. In a comparison between the maximum DLS-based performance (at length 6) with the maximum baseline performance (at length 2), DLS showed significant improvements over the baseline (z = 2.47, p < 0.01, jackknife resampled, one-tailed test).

### Long term stability

20 additional subjects were tested again 1 week later to evaluate the temporal stability of individual random generation behaviour. Again, we used jackknife resampling to estimate the cross pattern-length grand average identification performance statistics and compared the 40 min interval (128 subjects) and the 1 week interval (subset of 20 subjects). Identification performance after 1 week was qualitatively similar to the Identification rate after 40 min results, albeit marginally lower for all pattern lengths (see Fig. 2B). In our statistical analysis, this decrease in identification performance over time was not statistically significant (jackknife resampled linear model, z = 0.87, p = 0.62,), though our analysis may have been underpowered to detect such effects.

### Preferences and inhibitions

**Figure 3:**
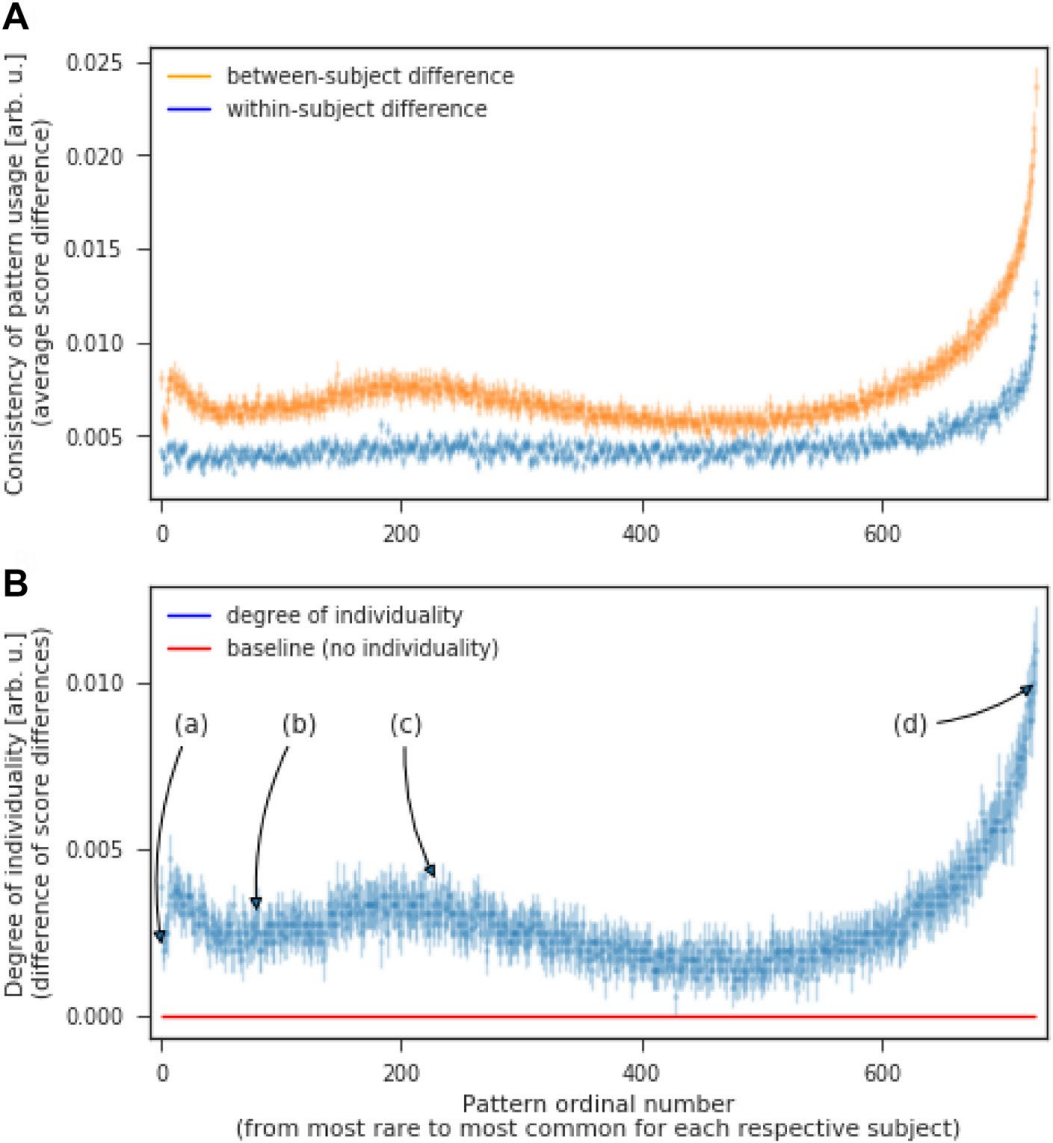
Subject specificity of rare vs. common patterns. (**A**) Inter- and intra-subject difference in usage of all 9^3^ length-3 patterns. The ordinal numbers represent each subject’s respective most rare to most common patterns. (**B**) The difference between inter- and intra-subject differences represents the degree of individuality for the respective rare and common patterns. The marked areas represent (a) universal exceptions, (b) rare patterns, (c) individual inhibitions, (d) individual preferences. All error bars represent SEM.

Finally, we investigated the mechanisms behind the intrinsic subject specificity of the random number sequences. Is the effect driven by individual preferences, individual pattern inhibitions, or perhaps both? Given the high identification performance, it is reasonable to assume that different subjects preferred (inhibited) different patterns. It thus does not help to examine specific patterns on the group level. Instead, for each subject, we found the most preferred (most prevalent) pattern in her first sequence, then calculated the difference in scores (prevalence) between (a) this pattern in her first sequence and this pattern in her second sequence (within-subject difference) and (b) this pattern in her first sequence and this pattern in all other subjects' sequences (between-subject difference). We repeated this process for the next most preferred patterns, then the next, etc., until we got to the most inhibited (most rare) patterns. In the end, we have a list of within-subject and between-subject differences for each subject's respective most inhibited and most preferred patterns. The difference between between-subject and within-subject difference represents the degree of individuality of a pattern (Fig. 3). This information was (implicitly) used by the algorithm in the last analysis to identify subjects based on their random sequences.

Five areas of interest can be identified: (a) *universal exceptions* are patterns no one expected in an RNG series (triplets of the same digit); (b) *rare patterns* are duplets and x–y–x patterns; their degree of individuality is dominated by individual pattern preferences; (c) *individual inhibitions* are subjectively perceived as non-random (these might potentially be birthdates, anniversaries, phone numbers—numbers we are too familiar with for them to feel random); (d) *individual preferences* that are person specific and that drive RNG identification.

To verify this interpretation we split the ordinal scale of length-3 patterns into 4 bands (Table 2, column 1) based on the local minima in Fig. 3B and extract the most common patterns in the respective areas (Table 2, column 2).

**Table 2:** Top-5 most common patterns in respective areas.

(a) universal exceptions [;10]	(6, 6, 6), (4, 4, 4), (8, 8, 8), (2, 2, 2), (3, 3, 3)
(b.1) rare patterns, duplets [11;200]	(4, 4, 5), (8, 8, 3), (9, 9, 4), (8, 8, 2), (8, 8, 7)
(b.2) rare patterns, xyx [201:230]	(6, 5, 6), (3, 2, 3), (9, 8, 8), (7, 5, 7), (8, 3, 6)
(c) individual inhibitions [230;300]	(1, 6, 8), (8, 4, 6), (3, 8, 4), (6, 7, 2), (4, 8, 1)
(d) individual preferences [700;]	(5, 7, 9), (3, 2, 1), (7, 9, 8), (3, 2, 5), (7, 8, 9)

### Cognitive capacity and environmental vs. procedural fingerprints

Globally, there are three possible explanations for individual pattern preferences and inhibitions. Firstly, the individuality might be due to differences in cognitive capacity: this is unlikely, as the few relevant components of cognitive capacity (e.g. working memory) should only affect correspondingly few dimensions of randomness. It cannot explain the entire effect of cognitive individuality. Secondly, preferences and inhibitions could be familiar patterns from the subjects personal environment: based on the degree of familiarity, subjects might either perceive these patterns as non-random and suppress them, or habitually produce them preferentially (“*environmental cognitive fingerprint*”). To investigate this, we tested whether each individual's familiar patterns (birth dates, postal codes, telephone numbers) were over- (or under-) represented in her sequence in comparison to this pattern’s prevalence in sequences from other subjects. Neither birth dates (t(114) = 0.22; p = 0.83/K–S for normality D = 0.05; p = 0.90), telephone numbers (t(19) = − 0.68; p = 0.50/D = 0.14; p = 0.89), nor postal codes (t(19) = − 1.19; p = 0.25/D = 0.13; p = 0.90) yielded significant results. Birth dates were collected ab inito for all subjects; 20 sets of telephone numbers and postal codes were collected post hoc for this experiment. Based on these results, we conclude that individuality in RNG is most likely the direct result of an individual generative algorithm (“*procedural cognitive fingerprint*”).

## Discussion

In the present study we address interindividual differences in the cognitive process of random number generation. We conclude that the mechanism by which humans generate random sequences is (a) highly unique and that (b) this uniqueness is driven by both individual preferences and individual inhibitions. This insight begs further questions: (a) do we really see unique generative algorithms or can our results be explained by individual differences in cognitive capacity? The latter is unlikely, as the few relevant components of cognitive capacity (e.g. working memory) should only affect correspondingly few dimensions of randomness. It cannot explain the entire effect of cognitive individuality. (b) Where do the individual preferences and inhibitions originate? They could be familiar patterns from the subjects personal environment: based on the degree of familiarity, subjects might either perceive these patterns as non-random and suppress them, or habitually produce them preferentially (“*environmental cognitive fingerprint*”). But tests for the individual telephone numbers, postal codes, and birthdates revealed no significant anomalies, leading us to conclude that individuality in RNG is most likely the direct result of an individual generative algorithm (“*procedural cognitive fingerprint*”).

Remarkably, single digit preferences alone allowed for identification of participants based on their random sequences. Individual digit preferences shine through in the random sequences, even though participants were instructed to generate an equal distribution of digits. Still, increasing the analyzed pattern length significantly improved the identification performance. Quantifying and delineating the contributions of different pattern lengths is beyond the scope of this manuscript and will require further research.

We were able to demonstrate that individual idiosyncrasies within the number sequence lead to robust identification performance and that individuality of the cognitive processes responsible for random number generation remains stable over at least a week. Further research is needed to explore the stability of patterns in individual random number generation behaviour on a larger number of participants over a longer test–retest interval.

Our results suggest that a tolerance to edit operations is necessary to leverage information for patterns longer than two digits. This reliance on tolerance to edit operations can be explained in two ways.

First, in the context of the generative processes for random sequences. In Baddeley’s model of random number generation^2,17^ participants use generative schemata to produce new responses, while continuously monitoring each new response for perceived randomness. In case of an unsatisfactory response, the response is inhibited and another schema is chosen. Consider that tolerance to edit operations as implemented by the DLS helps to extract predictive^9^ and identifying information. The implication for Baddeley’s model would be an extra step in which participants avoid switching between generative schemata by obscuring the unsatisfactory response via insertions, deletions, or transpositions.

The second possible explanation for the effectiveness of the DLS is its versatility. The DLS can be thought of as a generalized approach, incorporating many aspects of the established measures of randomness. For instance, the length-one DLS reflects the redundancy R, the length-two DLS reflects Evan’s RNG score^13^ with some tolerance for edit operations, and length > 2 DLS reflects, amongst others, runs (numerically increasing patterns, with tolerance for insertions). As such, there is no competition between traditional measures of randomness which aggregate information about the sequence into one scalar and the DLS which primarily serves as to represent a sequence in a high dimensional vector space.

Our experimental and statistical techniques enable simultaneous tracking of inhibitory (individual pattern inhibitions) and generative (pattern preferences) performance. This may be a useful tool for investigating attention-deficit/hyperactivity disorder, pharmacological interventions, or consequences of sleep deprivation. Moreover, our findings underline the notion that human cognition may not only vary cross-culturally^18^, but that the cognitive strategies and approaches people take to solve specific problems may be different from individual to individual.

## Supplementary Information

Supplementary Information.

## Data Availability

All statistical analyses were performed in Python. Scripts and custom software modules that were used to generate the results will be made accessible online for reproducibility and reuse at http://github.com/maschulz/rnglib.
