# Supplementary material for: A cognitive fingerprint in human random number generation
Source: Sci Rep. 2021 Oct 12;11:20217. doi: 10.1038/s41598-021-98315-y (PMC8511021; doi:10.1038/s41598-021-98315-y)
Supplement: Supplementary file 1 — Supplementary Information. [file 41598_2021_98315_MOESM1_ESM.pdf]

A COGNITIVE FINGERPRINT IN HUMAN RANDOM NUMBER GENERATION

SUPPLEMENTARY MATERIAL

Marc-André Schulz[1,*], Sebastian Baier[2], Benjamin Timmermann[2], Danilo Bzdok[3], and Karsten Witt[4]

[1]Department of Psychiatry and Psychotherapy, Charité – Universitätsmedizin Berlin, Berlin, Germany
[2]Department of Neurology, Christian-Albrecht-University Kiel, Kiel, Germany
[3]Department of Biomedical Engineering, Faculty of Medicine, McGill University, Montreal, Canada
[4]Department of Neurology and Research Center Neurosensory Science, Carl von Ossietzky University, Oldenburg, Germany
[*]Correspondence: marc-andre.schulz@charite.de

RNGT Instructions - translated from German, based on Towse (2007)

(a) Your task is to produce numbers in a random order. I shall tell you shortly which numbers to use.
(b) To give you an idea of what the task requires, imagine you roll a fair dice. Each side of the dice is equally likely to be selected with every roll, and each roll is independent of the preceding ones. I would like you to attempt to produce a set of numbers as if you were simulating a fair dice.
(c) Alternatively, imagine picking digits out of a hat. You pick one, read it out loud, put it back into the hat and pick another one, etc., etc..
(d) Try to make the sequence of numbers as unpredictable or as jumbled up as possible.
(e) You will hear a series of tones at the rate of 1 per second. Please produce a number from the range 1-9 (inclusive) each time you hear the computer give a signal, and continue until told to stop (this will be after ca. 5 minutes).
(f) The important part of the task is to keep pace with the tones, to give a number at the right time. Remember, there is no "right" or "wrong" answer to give, so there is no need to be anxious. If you miss a response do not stop, but continue naturally.
(g) Just try to produce a random sequence of numbers as best as you can.

a,b,e,f: from Towse (2007), Appendix 1;
paragraph on adjacent pairs and repeats removed for simplicity;
c: added the most commonly used analogy to explain the concept of randomness, see Baddeley (1966);
e: changed pace to 1Hz, see Daniels et al. (2003) vs. Towse (1998);
changed range from 1-10 to 1-9 for consistent use of only one-digit numbers;
changed length to time;
changed from 82 responses to 5 minutes (=300 responses)
f: added error handling

Procedure:

- Instructions
- Practice run
- 1. trial, 300 digits
- Pause / neuropsych. tests
- 2. trial, 300 digits
